# Opportunistic Screening for Low Bone Mineral Density in Adults with Cystic Fibrosis Using Low-Dose Computed Tomography of the Chest with Artificial Intelligence

**DOI:** 10.3390/jcm13195961

**Published:** 2024-10-07

**Authors:** Matthias Welsner, Henning Navel, Rene Hosch, Peter Rathsmann, Florian Stehling, Annie Mathew, Sivagurunathan Sutharsan, Svenja Strassburg, Dirk Westhölter, Christian Taube, Sebastian Zensen, Benedikt M. Schaarschmidt, Michael Forsting, Felix Nensa, Mathias Holtkamp, Johannes Haubold, Luca Salhöfer, Marcel Opitz

**Affiliations:** 1 Department of Pulmonary Medicine, Adult Cystic Fibrosis Center, University Hospital Essen-Ruhrlandklinik, University of Duisburg-Essen, 45239 Essen, Germany; 2 Department of Electrical Engineering and Applied Natural Sciences, Westphalian University of Applied Sciences, 45897 Gelsenkirchen, Germany; 3 Institute for Artificial Intelligence in Medicine, University Medicine Essen, 45147 Essen, Germany; 4 Department of Radiology, St. Josef Hospital Werden, University Medicine Essen, 45239 Essen, Germany; 5 Pediatric Pulmonology and Sleep Medicine, Cystic Fibrosis Center, Children’s Hospital, University of Duisburg-Essen, 45147 Essen, Germany; 6 Department of Endocrinology, Diabetes and Metabolism, Division of Laboratory Research, University Hospital Essen, 45147 Essen, Germany; 7 Institute of Diagnostic and Interventional Radiology and Neuroradiology, University Hospital Essen, University of Duisburg-Essen, 45147 Essen, Germany

**Keywords:** cystic fibrosis, adults, bone disease, osteoporosis, computed tomography, DXA, artificial intelligence, opportunistic screening, bone mineral density

## Abstract

**Background:** Cystic fibrosis bone disease (CFBD) is a common comorbidity in adult people with cystic fibrosis (pwCF), resulting in an increased risk of bone fractures. This study evaluated the capacity of artificial intelligence (AI)-assisted low-dose chest CT (LDCT) opportunistic screening for detecting low bone mineral density (BMD) in adult pwCF. **Methods:** In this retrospective single-center study, 65 adult pwCF (mean age 30.1 ± 7.5 years) underwent dual-energy X-ray absorptiometry (DXA) of the lumbar vertebrae L1 to L4 to determine BMD and corresponding z-scores and completed LDCTs of the chest within three months as part of routine clinical care. A fully automated CT-based AI algorithm measured the attenuation values (Hounsfield units [HU]) of the thoracic vertebrae Th9–Th12 and first lumbar vertebra L1. The ability of the algorithm to diagnose CFBD was assessed using receiver operating characteristic (ROC) curves. **Results:** HU values of Th9 to L1 and DXA-derived BMD and the corresponding z-scores of L1 to L4 showed a strong correlation (all *p* < 0.05). The area under the curve (AUC) for diagnosing low BMD was highest for L1 (0.796; *p* = 0.001) and Th11 (0.835; *p* < 0.001), resulting in a specificity of 84.9% at a sensitivity level of 75%. The HU threshold values for distinguishing normal from low BMD were <197 (L1) and <212 (Th11), respectively. **Conclusions:** Routine LDCT of the chest with the fully automated AI-guided determination of thoracic and lumbar vertebral attenuation values is a valuable tool for predicting low BMD in adult pwCF, with the best results for Th11 and L1. However, further studies are required to define clear threshold values.

## 1. Introduction

Cystic fibrosis (CF) is a rare autosomal recessive disease with serious chronically debilitating morbidities. CF is caused by a decreased quantity and/or function of the cystic fibrosis transmembrane regulator (CFTR) protein [1]. The CFTR is an ion channel that regulates the flow of chloride and other ions across the epithelia in various tissues, including the lungs, pancreas, other gastrointestinal organs, and sweat glands. Decreased CFTR quantity or function results in failure to regulate ion transport in these tissues, leading to a multisystem pathology associated with CF [2].

First described in 1979, bone disease is a well-recognized comorbidity in people with cystic fibrosis (pwCF), and its prevalence is increasing in the aging CF population [3,4]. Low bone mineral density (BMD) can increase the risk of low-trauma bone fractures and may result in the development of postural deviations, such as hyperkyphosis and scoliosis, which can have negative consequences for cough and airway clearance and can also limit the effectiveness of chest physiotherapy, contributing to the development of pulmonary exacerbations [5,6]. Therefore, it is crucial to diagnose bone demineralization at an early stage to ensure the appropriate treatment, management, and prevention of low-trauma fractures.

Dual-energy X-ray absorptiometry (DXA) is considered the gold standard for the diagnosis of cystic fibrosis-related bone disease (CFBD) [7]. CFBD is defined as the presence of low BMD, expressed as height- and age-adjusted z-scores of less than −2.0 standard deviations (SDs) determined by DXA [8]. European cystic fibrosis bone mineralization guidelines recommend routine bone density scans every five years if the BMD z-score is >−1, every two years if the z-score is between −1 and −2, and every year if the z-score is below −2 in adult pwCF younger than 50 years of age [8]. Therefore, osteoporosis in children, adolescents, and adults with CF is defined as having a BMD Z score of <−2 and a significant (low-trauma) fracture history [8].

The etiology of CFBD is multifactorial and encompasses a wide range of factors, including exocrine pancreatic insufficiency, diabetes mellitus, chronic inflammation, sex hormone deficiency, vitamin D and vitamin K deficiency, calcium malabsorption, malnutrition, delayed puberty, use of exogenous glucocorticoids, physical inactivity, and the direct effect of CFTR dysfunction on bone cell activity [9,10]. Even with normal BMD as measured with DXA, pwCF exhibits imbalanced bone turnover that favors bone resorption, abnormal bone microstructure, and increased risk of fractures compared to people without CF [11,12,13].

Despite the known high prevalence of osteoporosis (23.5%) and osteopenia (38%) in adult pwCF, current screening methods for CFBD, such as DXA, are often limited due to factors, such as accessibility, cost, and the potential risk of additional radiation exposure [6]. The significance of these limitations becomes particularly relevant in the context of CF, considering its growing life expectancy and the subsequent increase in the prevalence of osteopenia and osteoporosis with advancing age [14]. Therefore, it is encouraging to explore and establish alternative screening procedures that can overcome these limitations and improve screening efficiency for the early detection and management of low BMD and prevention of low-trauma bone fractures.

Low-dose computed tomography (LDCT) of the chest is a widely used imaging technique for detecting structural lung damage and evaluating the progression of pulmonary disease in adult pwCF [15]. LDCT scans of the chest with and without artificial intelligence (AI) techniques have been explored as an opportunistic approach for assessing low BMD in various studies outside of CF [16,17,18,19,20,21]. Opportunistic screening (OS) describes a method that enables the extraction of useful information from pre-existing images or image stacks such as chest CT scans, which were originally obtained for purposes unrelated to the targeted information [22]. These studies explored the feasibility of using CT attenuation values (Hounsfield Units [HU]) as surrogate markers for BMD measured using DXA and showed their remarkable potential for opportunistic low BMD screening. Despite the promising potential of LDCT with AI for OS of low BMD, its application in pwCF remains relatively unexplored, particularly in the context of establishing dependable HU threshold values for this unique population. Research studies that focus on the CF population, for whom the early detection of low BMD could significantly impact management approaches and outcomes, are scarce, thereby revealing a significant gap in the current research and clinical practice landscape. The present study aims to investigate the feasibility of implementing a fully automated AI-assisted approach for screening CFBD using LDCT scans of the chest. This method aims to automatically measure the attenuation values of the thoracic (Th9–Th12) and the first lumbar vertebra (L1) during routine clinical practice and assess their usefulness in identifying CFBD in adult pwCF.

## 2. Methods

### 2.1. Study Design and Participants

**Figure 1:**
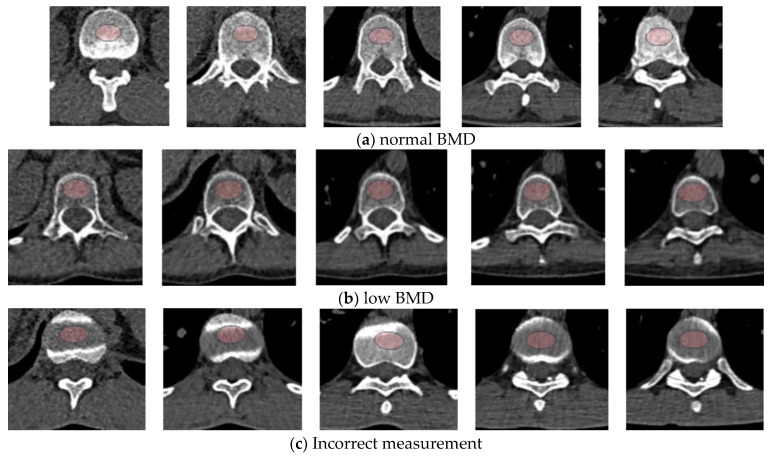
Examples of AI-assisted determinations of attenuation values for vertebral bodies L1 to Th9 using low-dose computed tomography (LDCT). From left to right: L1–Th9. (**a**) Normal findings. The ROI placed by the AI was appropriately centered on the vertebral body to be measured. The measured HU values ranged from 223 to 364. (**b**) Low BMD: The ROI is centered correctly by the AI on the vertebral bodies for the assessment of attenuation values. This results in HU values ranging between 89 and 117. (**c**) Incorrect measurements: The ROIs for L1, Th12, Th10, and Th9 were placed within the intervertebral disc space, resulting in underscored HU measurements for L1 (89), Th12 (122), Th10 (88), and Th9 (94). Conversely, the ROI for Th11 was positioned centrally within the vertebral body, with an HU value of 255. AI, artificial intelligence; ROI, region of interest; HU, Hounsfield unit; BMD, bone mass density.

This single-center retrospective study identified 71 pwCF aged 18–49 years from the Adult Cystic Fibrosis Center of Ruhrlandklinik Essen, Germany, who underwent DXA and LDCT of the chest within a 3-month period between April 2019 and May 2024, as part of routine clinical care. All subjects had a confirmed diagnosis of CF based on two disease-defining variants in their CFTR gene. Of the 71 pwCF, five patients were excluded from the study because of incorrect recognition of the region of interest (ROI) by AI (Figure 1), and one patient was excluded because of metal artifacts in the lumbar vertebrae that prevented DXA from being performed. After exclusion, 65 pwCF samples were available for analysis. Clinically relevant data, including pulmonary function test results, body mass index (BMI), 25-hydroxyvitamin D levels, and medication, were obtained from the electronic records nearest to DXA. None of the participants had a history of significant bone fracture. The duration between DXA and CT scan measurements was 6 ± 16 days.

This retrospective single-center study was approved by the local review board of the Essen University Hospital (no. 24-11977-BO) and followed the ethical principles of the Declaration of Helsinki for medical research involving human subjects.

### 2.2. Image Acquisition

#### 2.2.1. DXA Scanning

BMD was measured using a Horizon Ci scanner (Hologic Inc., Marlborough, MA, USA, software version 5.6) and a Lunar Prodigy scanner (GE Healthcare Technologies, Chicago, IL, USA). Routine lumbar anteroposterior scans were conducted with the BMD region of interest comprising the 1st to 4th lumbar vertebrae. Manufacturer reference values were used to determine the z-scores. A z-score of <−2 indicated low BMD.

#### 2.2.2. Computer Tomography and AI

All patients included in this study received non-contrast-enhanced LDCT scans of the chest using a 64-detector row single-source CT scanner (SOMATOM Definition AS, Siemens Healthcare GmbH, Forchheim, Germany) with a gantry rotation time of 300 ms (collimation: 64 × 0.6 mm; slice thickness: 1.0 mm; tube current-time product: 25 mAs; tube voltage: 120 kV). To optimize radiation protection, automatic tube current modulation (CARE Dose4D; Siemens Healthcare GmbH, Forchheim, Germany) and automatic tube voltage selection (CARE kV algorithm; Siemens Healthcare GmbH, Forchheim, Germany) were applied. Convolution kernels B31F and B70F were used for image reconstruction. The calibration of the CT system was performed quarterly using a standard water phantom provided by the manufacturer. In addition, daily calibration of the CT system was conducted using air.

Patients were scanned in an inspiratory breath-hold and head-first supine position with their arms elevated. For further image processing, reconstruction in the soft tissue window B31F with a 1 mm slice thickness was used for open-source Body and Organ Analysis (BOA), a comprehensive CT image segmentation algorithm designed for seamless workflow integration [23].

The open-source BOA integrates various segmentation algorithms with an emphasis on workflow integration through DICOM node integration, providing comprehensive body segmentation in CT images with extensive body volume coverage. The BOA incorporates two segmentation algorithms: Body Composition Analysis (BCA) and TotalSegmentator [24]. In this study, only BCA, which was trained using the nnU-Net framework, was used, as described by Haubold et al. [23].

The CT data were fully blinded, and BCA was used to automatically determine BMD from the available dataset. Based on HU, the following parameters for vertebrae Th9 to L1 were calculated: mean attenuation values with standard deviation and 95% confidence interval (CI). All the generated datasets were checked for quality. Inadequate ROI placement resulted in exclusion from the study (Figure 1).

### 2.3. Pulmonary Function Testing and Body Mass Index

BMI calculations and pulmonary function tests (PFTs) were obtained from the electronic records nearest to the DXA scans. A JAEGER MasterScreen body (CareFusion, Hoechberg, Germany) was used to measure forced expiratory volume in 1 s (FEV1). The Global Lung Function Initiative (GLI) reference values were used [25].

### 2.4. Statistical Analysis

Statistical analyses were performed using GraphPad Prism, version 10.2 (GraphPad Software, San Diego, CA, USA). Data are presented as the mean ± standard deviation. Normal distribution was tested using the Shapiro–Wilk test. Student’s *t*-test or Mann–Whitney U-test was used to assess differences between groups, as appropriate. The chi-square test (χ^2^) or Fisher’s exact test was used to assess the differences between categorical variables. Pearson’s correlation coefficient was used to measure the linear correlation between two sets of metric data. Receiver operating characteristic (ROC) curve analysis was performed to identify predictors of low BMD using AI-determined HU values of vertebrae Th9 to L1. ROC curve plotting and ROC analysis, including the determination of the area under the ROC curve (AUC), specificity, and sensitivity for each variable, and determination of thresholds, were calculated. Statistical significance was set at *p* < 0.05.

## 3. Results

### 3.1. Study Population

A total of 65 pwCF were included in this study, of which there were 25 females (38%). The majority of the study population was homozygous for F508del, accounting for 46% of the total, with an average age of 30.1 ± 7.5 years. The mean ppFEV1 was 58.8 ± 21.7, and the BMI ranged from 14.0 to 39.0 kg/m^2^, with a mean of 21.8 ± 4.0. Exocrine pancreatic insufficiency was identified in 53 of 65 pwCF (82%), whereas CF-related diabetes mellitus was present in 16 patients (25%). The 25-hydroxyvitamin D levels ranged from 4.3 to 54.2 ng/mL, with 77% receiving oral cholecalciferol replacement therapy. Table 1 presents the demographic and clinical characteristics of the study population.

### 3.2. Dual-Energy X-ray Absorptiometry (DXA)

Twelve participants (18%) exhibited low BMD for age on the DXA scan (L1-L4) with a z-score of <−2, as shown in Table 2. The overall mean bone mineral density was 0.97 ± 0.15 g/cm^2^, corresponding to a z-score of −1.0 ± 1.3. Statistically significant differences were observed between pwCF with normal BMD and those with low BMD in terms of ppFEV1 (*p* = 0.019) and BMI (*p* = 0.020). No other clinical characteristics were significantly different between the two groups (Table 1).

### 3.3. CT-Derived Bone Density

Table 2 shows AI-calculated CT-derived attenuation values for Th9 to L1, with L1 consistently exhibiting the lowest HU values among the examined vertebral bodies. Patients with DXA-determined low BMD demonstrated significantly lower HU values in all examined vertebral bodies, except for Th12 (*p* = 0.055), compared to those without bone disease (all *p* <0.05).

### 3.4. Correlation between DXA (Z-Scores and BMD) and CT-Derived Bone Density

**Figure 2:**
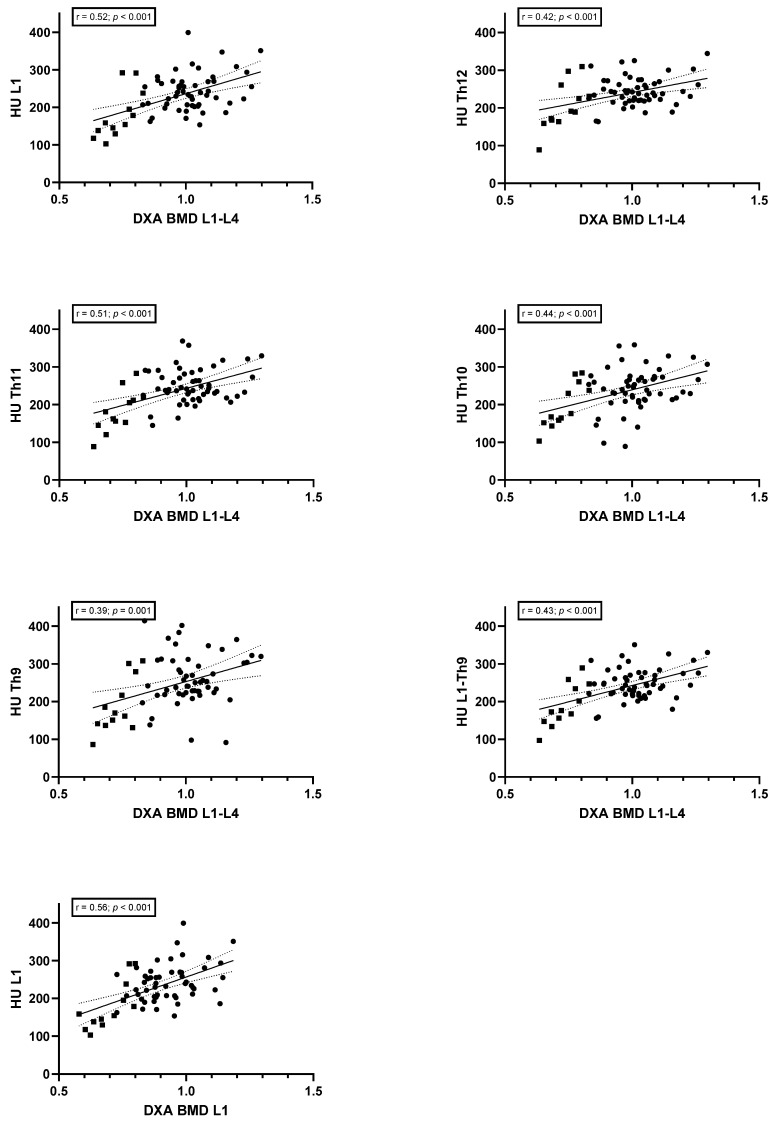
Scatter plot of CT-attenuation values and DXA-derived BMD. •, normal BMD; ▪, CFBD. CFBD, cystic fibrosis-related bone disease; HU, Hounsfield units; DXA, dual-energy X-ray absorptiometry; BMD, bone mineral density; r, correlation coefficient.

**Figure 3:**
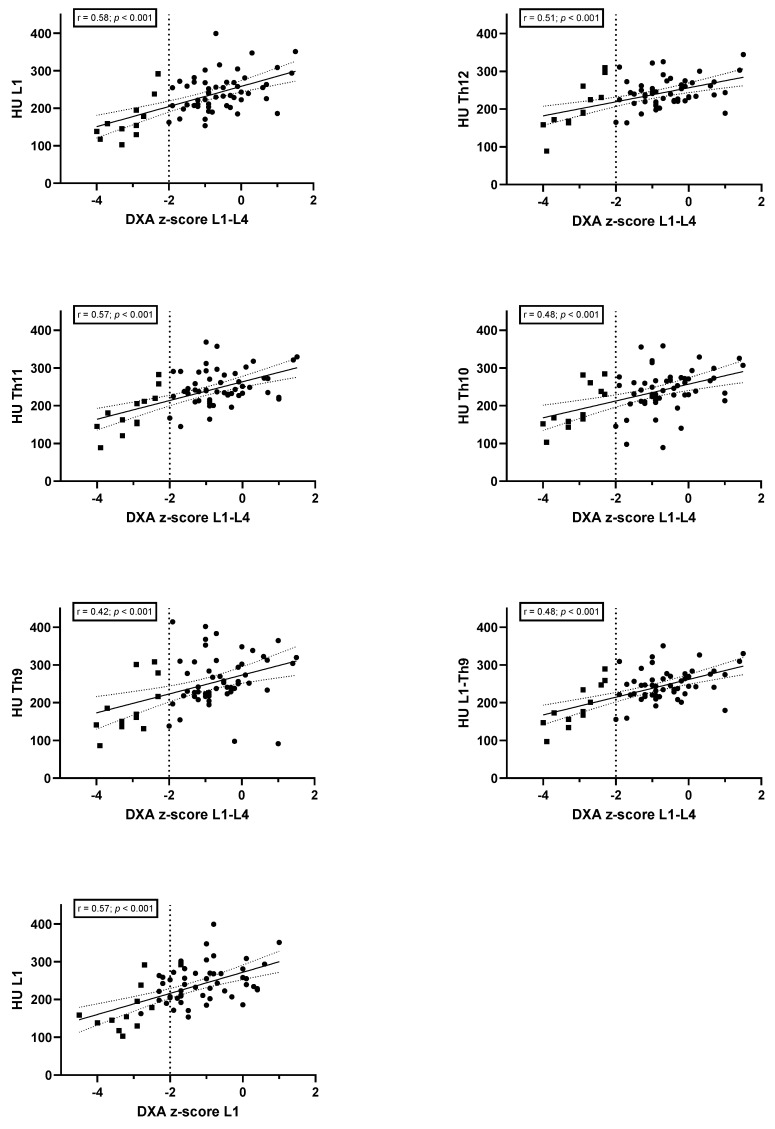
Scatter plot of CT-attenuation values and DXA-derived z-scores. •, normal BMD; ▪, CFBD CFBD, cystic fibrosis-related bone disease; HU, Hounsfield units; DXA, dual-energy X-ray absorptiometry; BMD, bone mineral density; r, correlation coefficient.

The findings of the correlation analysis are presented in Figure 2 and Figure 3, respectively. The analysis showed a positive association between the AI-estimated HU values and the DXA-derived BMD and z-scores for L1–L4. The strongest correlations were identified between L1 and BMD (r = 0.52; *p* < 0.001) and z-scores (r = 0.58; *p* < 0.001). A correlation analysis conducted independently on L1, as determined by LDCT and DXA, revealed a significant association for both BMD (r = 0.56; *p* < 0.001) and z-score (r = 0.57; *p* < 0.001). However, the mean HU values of Th9 to L1 did not exhibit improved results when compared with DXA (r = 0.43, *p* < 0.001 for BMD and r = 0.48, *p* < 0.001 for z-score).

### 3.5. ROC Analysis

**Figure 4:**
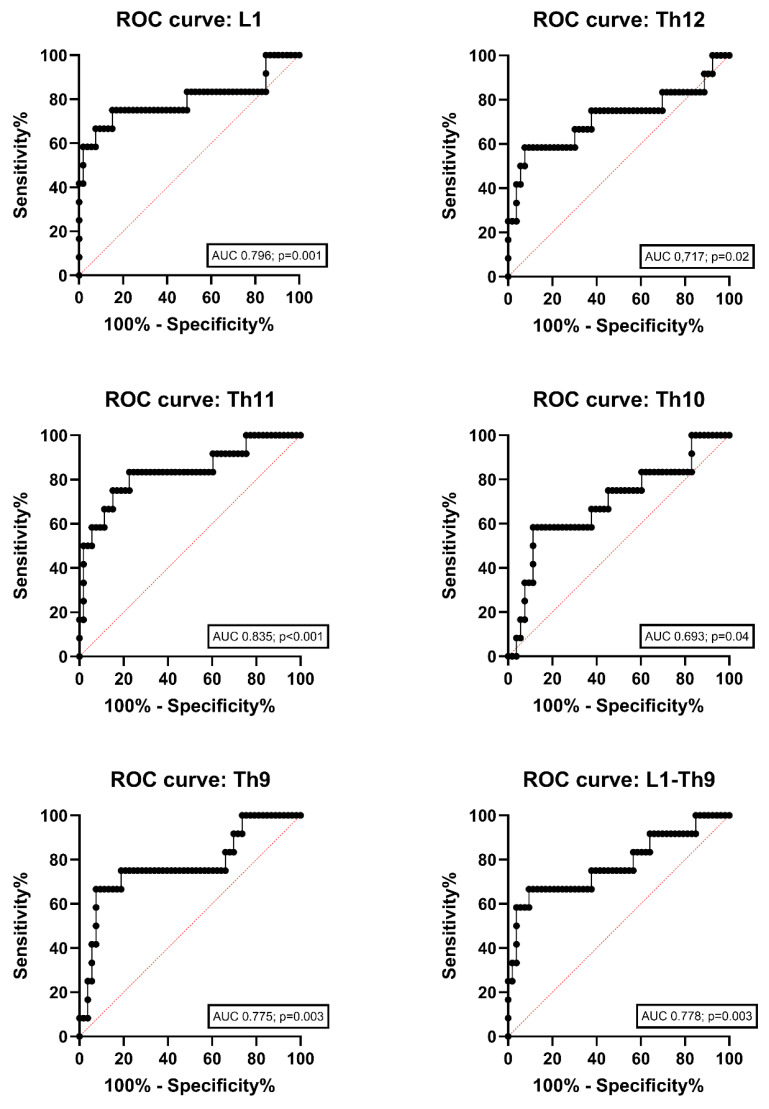
Receiver operating characteristic (ROC) curves of CT-attentuation values for predicting low BMD. AUC, area under the curve; BMD, bone mineral density; ROC, receiver operating characteristic.

The results of the ROC analysis of AI-estimated attenuation values revealed that Th11 and L1 demonstrated the highest AUC values for distinguishing between the low BMD and normal group. Th11 had an AUC value of 0.835 (*p* < 0.001), whereas L1 had an AUC value of 0.796 (*p* = 0.001) (Figure 4). Table 3 shows the HU thresholds at a sensitivity level of 75% for each vertebra. Th11 and L1 exhibited the highest specificity for differentiating between pwCF with low BMD and those with normal BMD at a sensitivity level of 75%, with thresholds of <212 HU and <197 HU, respectively, and a specificity of 84.9% for both.

## 4. Discussion

Our study indicates that the LDCT of the chest using a fully automated AI-guided determination of Th9 to L1 trabecular attenuation values has promising potential for opportunistic screening of low BMD in adult pwCF. Our findings revealed a positive correlation between the HU values of Th9 to L1 and both DXA-derived z-scores and BMD of L1 to L4. Moreover, we established threshold values for Th9 to L1 with acceptable specificity and sensitivity levels for diagnosing low BMD, with the best results achieved for Th11 and L1.

The growing significance of the early diagnosis of comorbidities in pwCF is noteworthy, considering the increasing life expectancy and subsequent increase in comorbidities [3]. In addition to CF, AI has been investigated for its potential to enhance OS for low BMD during routine non-enhanced LDCT scans of the chest by leveraging existing imaging resources to identify individuals at risk of osteoporosis [20]. This method has the potential to improve screening rates, reduce costs and radiation dose, and facilitate early intervention, particularly in settings where access to DXA is limited or in high-risk populations such as CF, which may benefit from routine BMD assessment [26,27].

It is difficult to compare our data with those of existing studies. The outcomes of AI-assisted LDCT scanning for low BMD screening vary considerably based on factors such as the scanned region, the patient population being examined, and the specific CT scanner and AI software used. A meta-analysis conducted by Ong et al. revealed a sensitivity of 41.0–100% and a specificity of 31.0–100%, which reflects the great heterogeneity of the AI techniques used and the examined populations for differentiating between normal and low BMD [28]. With respect to the disease, our study cohort was much younger than those used in other studies for assessing opportunistic low BMD screening with LDCT. These studies predominantly involved older adults, often postmenopausal women or patients aged >50 years, with varying disease conditions, reflecting the higher prevalence and clinical concern of low BMD in these groups [17]. Normative attenuation values for L1 and Th9–12 in young adults can be inferred from the data provided by Jang et al. [29] and Patel and Lee [30]. Looking at L1, Jang et al. reported a mean L1 attenuation of 226 ± 44 HU in patients younger than 30 years and 192 ± 39 HU in patients 45–49 years of age. These results are in line with ours, showing overall HU values for L1 of 230 ± 58. Patel and Lee suggested that young adults typically exhibit higher HU values at every spine level than older age groups, where the mean attenuation values of the spine decrease linearly with age [30].

Therefore, determining HU thresholds to differentiate normal from low BMD is challenging and varies considerably across the literature. Research conducted by Yang et al. established cutoff values for distinguishing between normal and low BMD, with values ranging from 147.5 (L1) to 168.5 (Th9) [20]. The current study reported a high diagnostic efficiency for AI-based screening, with AUC values ranging from 0.772 to 0.793 (Th9–L1), which is consistent with the AUC values in our study (0.693–0.835). In a literature review by Zaidi et al. an L1–HU value of 135 was defined as the limit for determining low BMD [31]. However, our study identified higher threshold values for single vertebrae to differentiate between normal and low BMD, mainly because of the younger age of the studied population (Table 3). This highlights the complexity involved in setting the threshold values and emphasizes the dependence of these values on the patient population being studied.

Although the use of fully automated AI-guided LDCT for the opportunistic screening of low BMD is increasing, several factors should be considered. Automated segmentation plays a key role in the accurate identification of bone and vertebral levels [32]. In many CT scans of the chest, the vertebra of interest is not fully captured and may be at the edge of the field of view, leading to partial inclusion, which can lead to the over- or underestimation of the HU values. However, spinal deformities such as scoliosis or kyphosis, which are prevalent in pwCF, can complicate the automatic detection of vertebral bodies [5]. The accurate identification of spinal levels is essential because research indicates that BMD varies among different spinal levels and may lead to the incorrect determination of attenuation values [30].

DXA is recognized as a precise method for assessing BMD yet presents certain limitations. Inaccurate positioning, false scan mode, artifacts, and spinal deformations may affect the accuracy of BMD determination [33].

### Limitations

First, the retrospective design of this study, which involved individuals who underwent paired chest CT scans and DXA examinations at a three-month interval, may have resulted in selection bias and could potentially limit the generalizability of the findings. Furthermore, the small number of patients, particularly those with confirmed osteoporosis, may have influenced the determination of the reference values and led to over- or underestimation. Additionally, the AI-supported recognition of vertebral bodies for determining HU may be faulty (Figure 1), which could result in the under-sharpening or over-sharpening of the measurements, directly affecting the diagnostic outcome. It must also be noted that the definition of low BMD or osteoporosis in pwCF differs from the World Health Organization (WHO)’s definition for assessing bone density, which is employed in the majority of studies on opportunistic CT screening [34]. This variance may introduce potential bias when comparing our findings with those of other researchers. The accuracy of AI models is high; however, they require validation against large, diverse datasets before integration into routine clinical workflows. Finally, our data do not provide information regarding the potential fracture risk in pwCF DXA-measured BMD as it does not correlate well with fracture risk in pwCF [11]. Additional longitudinal data are necessary to determine whether the overall prediction of fracture risk using LDCT of the chest with and without AI will be able to address this limitation in adults with CF.

## 5. Conclusions

Fully automated AI-supported opportunistic screening for low BMD using LDCT scans of the chest shows great potential for use in adult pwCF. However, future research is needed to determine the diagnostic accuracy of this method and to establish clear thresholds to effectively differentiate between normal and low BMD for the diagnosis of CFDB and assessment of potential bone fracture risk in adults with CF.

## Figures and Tables

**Table 1:** Patient demographics and characteristics.

Characteristics	Overall (*n* = 65)	Normal (*n* = 53)	CFBD (*n* = 12)	*p*-Value (normal vs. CFBD)
Age, years	30.1 ± 7.5 (18–49)	30.2 ± 7.8 (18–49)	29.8 ± 6.5 (20–42)	0.820
Female sex, n (%)	25 (38)			0.754
Genotype, n (%)				0.598
F508del homozygous	30 (46)	24 (45)	6 (50)	
F508del heterozygous	23 (36)	18 (34)	5 (42)	
Other	12 (18)	11 (21)	1 (8)	
CFTR modulator therapy, n (%)				0.470
Elexacaftor/tezacaftor/ivacaftor	18 (28)	13 (25)	5 (42)	
Tezacaftor/ivacaftor	6 (9)	4 (8)	2 (17)	
Lumacaftor/ivacaftor	3 (5)	3 (6)	0	
Ivacaftor	2 (3)	2 (4)	0	
None	36 (55)	31 (58)	5 (42)	
ppFEV1	58.8 ± 21.7 (21–120)	61.4 ± 22.6 (21–120)	47.3 ± 12.6 (29–68)	0.006
FEV1 [L]	2.4 ± 1.1 (0.7–4.9)	2.5 ± 1.1 (0.7–4.9)	1.9 ± 0.5 (1.2–3.0)	0.006
BMI [kg/m^2^]	21.8 ± 4.0 (14.0–39.0)	22.4 ± 4.0 (14.9–39.0)	19.4 ± 3.3 (14.0–24.6)	0.020
Pancreatic insufficiency, n (%)	53 (82)	42 (80)	11 (92)	0.317
Cystic fibrosis-related diabetes, n (%)	16 (25)	15 (28)	1 (8)	0.147
25-hydroxyvitamin D levels [ng/mL] ^#^	22.0 ± 9.5 (4.3–54.2)	21.6 ± 7.6 (6.9–38.8)	23.7 ± 15.7 (4.3–54.2)	0.660
Cholecalciferol supplementation, n (%)	50 (77)	39 (74)	11 (92)	0.179
Steroid therapy, n (%)	5 (8)	4 (8)	1 (8)	0.926

Values are the mean ± standard deviation, range, or number of patients (%). CFTR, cystic fibrosis transmembrane conductance regulator; ppFEV1, percent-predicted forced expiratory volume in 1 s; BMI, body mass index; CFBD, cystic fibrosis-related bone disease. # n = 64.

**Table 2:** Results of DXA and CT-attenuation values measurement.

Characteristics	Overall (*n* = 65)	Normal (*n* = 53)	CFBD (*n* = 12)	*p*-Value (Normal vs. CFBD)
DXA z-score L1-L4	−1.0 ± 1.3 [−1.35–−0.73]	0.6 ± 0.8 [−0.81–−0.35]	−3.0 ± 0.6 [−3.43–−2.67]	<0.001
DXA BMD L1-L4 [g/cm^2^]	0.97 ± 0.15 [0.93–1.01]	1.02 ± 0.11 [0.99–1.05]	0.73 ± 0.06 [0.69–0.77]	<0.001
DXA z-score L1	−1.5 ± 1.2 [−1.77–−1.19]	−1.1 ± 0.9 [−1.36–−0.86]	−3.1 ± 0.7 [−3.59–−2.66]	<0.001
DXA BMD L1 [g/cm^2^]	0.89 ± 0.14 [0.86–0.92]	0.93 ± 0.11 [0.90–0.96]	0.70 ± 0.08 [0.65–0.75]	<0.001
HU Th9	247.1 ± 73.9 [228.79–265.40]	260.3 ± 68.3 [241.44–279.10]	188.9 ± 72.1 [143.09–234.75]	0.002
HU Th10	234.0 ± 58.7 [219.41–248.50]	242.4 ± 55.6 [227.07–257.76]	196.6 ± 60.0 [158.75–234.46]	0.013
HU Th11	237.7 ± 54.2 [224.30–251.18]	250.4 ± 45.6 [237.79–262.93]	182.0 ± 56.0 [146.36–217.56]	<0.001
HU Th12	237.0 ± 45.8 [225.71–248.42]	244.5 ± 37.9 [234.0–254.91]	204.4 ± 63.2 [164.26–244.58]	0.055
HU L1	230.4 ± 57.6 [216.15–244.71]	242.2 ± 49.5 [228.54–255.87]	178.4 ± 64.0 [137.74–219.07]	<0.001
HU Th9–L1	237.3 ± 49.7 [224.94–249.57]	247.1 ± 43.4 [235.13–259.01]	193.9 ± 54.8 [159.0–228.76]	0.001

Values are presented as the mean ± standard deviation. Brackets indicate the 95% CI. CI, confidence interval; DXA, dual-energy X-ray absorptiometry; BMD, bone mineral density; HU, Hounsfield units; CFBD, cystic fibrosis-related bone disease.

**Table 3:** Sensitivity and specificity and likelihood ratio of different density thresholds for Th9 to L1 indicating low BMD.

HU Threshold		Sensitivity (%)	95% CI	Specificity%	95% CI	Likelihood Ratio
<190	Th9	66.7	39.1–86.2	92.5	82.1–97.0	8.8
<217	Th9	75.0	46.8–91.1	81.1	68.6–89.4	4.0
<185	Th10	58.3	32.0–80.7	88.7	77.4–94.7	5.2
<238	Th10	75.0	46.8–91.1	54.7	41.5–67.3	1.7
<163	Th11	50.0	25.4–74.6	98.1	90.1–99.9	26.5
<212	Th11	75.0	46.8–91.1	84.9	72.9–92.1	5.0
<179	Th12	41.7	19.3–68.0	96.2	87.2–99.3	11.0
<232	Th12	75.0	46.8–91.1	62.3	48.8–74.1	2.0
<160	L1	58.3	19.3–68.0	98.1	90.1–99.9	30.9
<197	L1	75.0	46.8–91.1	84.9	72.9–91.1	5.0
<178	Th9 -L1	58.3	32.0–80.7	96.2	87.2 to 99.3%	15.5
<234	Th9 -L1	75.0	46.8–91.1	62.3	48.8 to 74.1%	2.0

BMD, bone mineral density; CI, confidence interval; HU, Hounsfield units.

## Data Availability

Data used to support the current findings are available from the corresponding author upon request.

## Abbreviations

AI	artificial intelligence
AUC	area under the curve
BCA	body composition analysis
BMI	body mass index
BOA	body organ analysis
CF	cystic fibrosis
CFBD	cystic fibrosis-related bone disease
CFTR	cystic fibrosis transmembrane conductance regulator
CT	computed tomography
DXA	dual-energy X-ray absorptiometry
LDCT	low-dose computed tomography
PFT	pulmonary function testing
ppFEV_1_	percent predicted forced expiratory volume in 1 s
pwCF	people with cystic fibrosis
ROC	receiver operating characteristic
